# 

**Published:** 1867-06

**Authors:** 


### CONTENTS FOR JUNE.

ORIGINAL CONTRIBUTIONS.

Ovariotomy. By S. D. Jackson, M.D., Chicago, Ill. ........ 241
Atomizing Instruments. By Benjamin Durham, Jr., M.D., ........ 245
Anterior Dislocation of the Elbow-Joint. By R. P. Hunt, M.D., Chicago. 251
Milk-Sickness. By L. P. Newman, M.D., Chicago, Ill ........ 253
Removal of Uterine Polypus by a Modified Form of the Ecraseur. By Jonathan W. Brooks, M.D., Chicago, Ill. ........ 254
The Endoscope, and its Application to the Diagnosis and Treatment of Urinary Affections. By A. J. Desormeaux, M.D., Translated by R. P. Hunt, M.D. Continued from Page 208 ........ 273

PROCEEDINGS OF SOCIETIES.

Chicago Medical Society ........ 258

EDITORIAL.—To Subscribers ........ 262
Book Notices ........ 262
Apologetic ........ 263
Thanks ........ 268
American Naturalist ........ 263
The Illinois Homœopathists ........ 263
American Medical Association ........ 264
The Chicago College of Pharmacy ........ 264
American Pharmaceutical Association ........ 265

MISCELLANEOUS.

To the American Medical Association ........ 266
Homœopathists vs. Homœopathy ........ 268
The Treatment of Cancer ........ 269
Incontinence of Urine Successfully Treated by Extract of Belladona ........ 271
The Ventilating Method ........ 272
Receipts Acknowledged ........ 272

